# Clinical burden, risk factor impact and outcomes following myocardial infarction and stroke: A 25-year individual patient level linkage study

**DOI:** 10.1016/j.lanepe.2021.100141

**Published:** 2021-06-16

**Authors:** Anoop S.V. Shah, Kuan Ken Lee, Jesús Alberto Rodríguez Pérez, Desmond Campbell, Federica Astengo, Jennifer Logue, Peter James Gallacher, Srinivasa Vittal Katikireddi, Rong Bing, Shirjel R. Alam, Atul Anand, Catherine Sudlow, Colin M Fischbacher, Jim Lewsey, Pablo Perel, David E. Newby, Nicholas L. Mills, David A. McAllister

**Affiliations:** a Department of Non-communicable Disease Epidemiology, London School of Hygiene and Tropical Medicine,London, United Kingdom; b Department of Cardiology, Imperial College NHS Trust, London, United Kingdom; c BHF Centre for Cardiovascular Science, University of Edinburgh, Edinburgh, United Kingdom; d Institute of Health and Wellbeing, University of Glasgow, Glasgow, United Kingdom; e Lancaster University, Lancaster, United Kingdom; f Usher Institute, University of Edinburgh, Edinburgh, United Kingdom; g Public HealthScotland, United Kingdom

## Abstract

**Background:**

Understanding trends in the incidence and outcomes of myocardial infarction and stroke, and how these are influenced by changes in cardiovascular risk factors can inform health policy and healthcare provision.

**Methods:**

We identified all patients 30 years or older with myocardial infarction or stroke in Scotland. Risk factor levels were determined from national health surveys. Incidence, potential impact fractions and burden attributable to risk factor changes were calculated. Risk of subsequent fatal and non-fatal events (myocardial infarction, stroke, bleeding and heart failure hospitalization) were calculated with multi-state models.

**Findings:**

From 1990 to 2014, there were 372,873 (71±13 years) myocardial infarctions and 290,927 (74±13 years) ischemic or hemorrhagic strokes. Age-standardized incidence per 100,000 fell from 1,069 (95% confidence interval, 1,024-1,116) to 276 (263-290) for myocardial infarction and from 608 (581-636) to 188 (178-197) for ischemic stroke. Systolic blood pressure, smoking and cholesterol decreased, but body-mass index increased, and diabetes prevalence doubled. Changes in risk factors accounted for a 74% (57-91%) reduction in myocardial infarction and 68% (55-83%) reduction in ischemic stroke. Following myocardial infarction, the risk of death decreased (30% to 20%), but non-fatal events increased (20% to 24%) whereas the risk of both death (47% to 34%) and non-fatal events (22% to 17%) decreased following stroke.

**Interpretation:**

Over the last 25 years, substantial reductions in myocardial infarction and ischemic stroke incidence are attributable to major shifts in risk factor levels. Deaths following the index event decreased for both myocardial infarction and stroke, but rates remained substantially higher for stroke.

**Funding:**

British heart foundation

**Research in Context**

**Evidence before this study**

We searched Ovid Medline (from 01/01/1995 to 01/08/2020) for search terms ‘cardiovascular risk factors’, ‘incidence’, ‘myocardial infarction’, ‘stroke’ and ‘outcomes’. We identified several studies including systematic reviews evaluating trends in incident myocardial infarction or stroke. Several studies evaluated impact of changes in risk factors on coronary death. We found no large contemporary nationwide studies comparing the clinical burden of stroke and myocardial infarction, evaluating the impact of population changes in risk factors on fatal and non-fatal disease and the short- and longer-term fatal and non-fatal clinical sequalae following index event.

**Added value of this study**

In a large contemporary nationwide patient level linkage study we show that substantial reductions in myocardial infarction and ischemic stroke incidence are associated with reductions in systolic blood pressure, smoking and cholesterol but attenuated by increases in body mass index and diabetes prevalence. Importantly, we now show that in addition to the incidence of both myocardial infarction and stroke declining, the temporal difference in the incidence has also narrowed. Deaths following the index event decreased for both myocardial infarction and stroke, but rates remained substantially higher for stroke. The clinical burden of stroke, both in terms of incidence and case-fatality, now contributes to a greater burden of acute cardiovascular disease.

**Implications of all the available evidence**

Substantial reductions in the incidence of both myocardial infarction and ischemic stroke can be achieved by addressing biological and behavioural risk factors. These observations have major implications for countries in low- and middle-income settings where rates of hypertension and dyslipidaemia are rising as well as the residual risk from persistent uncontrolled risk factors in high-income countries. We also highlight the increasing influence of obesity and diabetes on acute cardiovascular events, which have implications for all countries given that both risk factors are rising worldwide. Finally, differences in the burden of myocardial infarction and stroke are narrowing, particularly in women, and monitoring these trends is essential for the planning of healthcare provision in the decades that follow.

Alt-text: Unlabelled box

## 1 Introduction

Planning of healthcare provision for acute cardiovascular disease requires an understanding of population trends in both the incidence and subsequent clinical outcomes following acute presentation. Insights into how these trends have been impacted by changes in risk factor profiles over time are needed to inform public health policy. Despite considerable overlap between myocardial infarction and stroke, with shared pathophysiological mechanisms, risk factors and management, they have rarely been studied concurrently.[1] Previous studies comparing incidence for these two conditions were not contemporary.[1], [2], [3] Those reporting trends for myocardial infarction have predominantly been limited to fatal events,[4], [5], [6], [7], [8], [9]whilst stroke studies have been limited to smaller cohorts and younger populations.[10,[11], [12], [13]] Whilst the associations between risk factors and myocardial infarction[14] or stroke[15,16] have been studied,the impact of temporal changes in risk factors on the incidence of both, and in those with and without fatal presentations, remains uncertain.

The last two decades has seen substantial changes in the management and outcomes of myocardial infarction and stroke.[12,[17], [18], [19], [20], [21], [22], [23]] Most studies have reported trends in morbidity and mortality during the acute phase,[13,24,25] with studies of the longer term sequelae scarce[26] and report inconsistent findings.[27], [28], [29], [30], [31]

Reliable information about the comparative epidemiology regarding clinical burden, risk factor impact and the subsequent sequelae following myocardial infarction and stroke remains scarce. To inform public health policy and healthcare provision, we investigated the impact of population changes in risk factors over 25 years on incident myocardial infarction and stroke. We also report on changes in risk of subsequent fatal events, and single and multiple non-fatal events following index non-fatal myocardial infarction or stroke.

## 2 Methods

### 2.1 Study population, design and data sources

An individual patient-level linkage study ***(Supplement figure 1)*** using national datasets in Scotland (***Supplementary text 1)*** included all patients 30 yearsof age or older with myocardial infarction or stroke from January 1^st^, 1990 to December 21^st^, 2014 with follow-up until December 31^st^, 2017. Using a 5-year lookback period ***(Supplementary text 2)***, index cases were identified as ICD-10 coded hospitalisations or deaths without prior hospitalisation***(Supplementary table 1).***Age at incident event, sex, comorbidity and area-based measure of deprivation using the Scottish Index of Multiple Deprivation (SIMD) were extracted.[32]

All index cases were linked to prior and subsequent hospital episodes and deaths from the National Health Service (NHS) Scottish Morbidity Register and National Records of Scotland using theCommunity Health Index, a register of all Scottish NHS patients. Age- and sex-stratified mid-year population estimates were obtained from National Records of Scotland. Non-fatal events consisted of heart failure, stroke, myocardial infarction and bleeding. Access to the data was approved by the NHS Scotland Public Benefit and Privacy Panel for Health and Social Care.

Age-, sex- and year-stratified population distribution of cardiovascular risk factors (systolic blood pressure, total cholesterol, body mass index and the prevalence of smoking and diabetes mellitus) were estimated from the Scottish Health Survey.[33] The Scottish Health Survey uses a multistage stratified clustered probability sampling design to ensure the results are representative of the population ***(Supplementary text 3)***. The surveys were conducted in 1995, 1998, 2003 and then annually from 2008. Age- and sex-stratified risk ratios for each risk factor were extracted from the published literature.[[14], [15], [16],^34^]

### 2.2 Statistical analysis

Baseline characteristics were summarised in 5-year intervals by index event.

*Incidence modelling:* Generalized additive models were used to estimate trends in incident myocardial infarction and stroke. Annual incident hospitalisations were aggregated by 10-year age intervals and sex separately for index myocardial infarction and stroke. Person-time, using the same covariates, was calculated using the population mid-year estimates ***(Supplementary text 2)***. For incidence rates, a log link and Poisson error distribution were used with a scaling factor (quasi-Poisson) to allow for overdispersion. Annual incidence rates were standardized to the European Standard Population.[35]

*Burden:* For each risk factor, regression models were constructed from baseline health survey data to estimate age-, sex- and year-stratified values ***(Supplement text 4)***. The number of events prevented, delayed or caused as a result of a change in risk factor level was estimated using data from three sources: the age-, sex- and year-stratified incident count, the age- and sex-stratified (where available) relative risk and absolute change in age- and sex-stratified risk factor level. Distributions were combined via Monte Carlo sampling to produce estimates of the potential impact fraction[36] (a measure of the proportional reduction in the disease or mortality risk, when the risk factor distributions change) and a final count distribution for each age-, sex- and year-strata of events prevented, delayed or caused by change in risk factor levels*(****Supplementary text 5)***.

*Multistate outcome models:* Time to subsequent fatal and non-fatal events (3-year follow-up) was modelled using multi-state models for index non-fatal myocardial infarction and stroke. Weibull accelerated failure time models were fitted, adjusting for age, sex, deprivation, cohort period and comorbidity **(Supplementary text 6)**. Estimated risks were presented at the population level stratified by cohort period and standardized using the count data of the remaining categorical variables (sex, deprivation and comorbidity) as well as by age, sex, deprivation, comorbidity and cohort period. Statistical analyses were performed in R version 3.5.1 (Vienna, Austria).

*Role of funding source:* Funders had no role in study design, data collection, data analysis, interpretation or writing of the report

## 3 Results

From 1990 to 2014, among 605,996 patients, there were 372,873 (71±13 years, 43% female) and 290,927 (74±13 years, 55% female) index myocardial infarctions and strokes respectively. Compared to stroke, patients with myocardial infarction were younger and more likely to be male*.*Across the cohort periods, there was a modest narrowing in social inequalities (Table 1***, Supplementary table 2 and 3***).

**Table 1.** Baseline characteristics in patients with index fatal and non-fatal myocardial infarction or stroke stratified by 5-year calendar groups.

Variable	Index Condition	1990 to 1994	1995 to 1999	2000 to 2004	2005 to 2009	2010 to 2014
Number	Myocardial infarction	100,929	82,330	69,415	58,513	61,686
	Stroke	64,870	64,593	58,563	52,043	50,858
Age, years (mean [SD])	Myocardial infarction	70.2 (12.2)	70.9 (12.5)	71.5 (13.1)	71.3 (13.6)	70.2 (13.9)
	Stroke	74.0 (12.2)	74.0 (12.6)	74.0 (13.1)	73.5 (13.5)	73.4 (13.6)
Sex, females(%)	Myocardial infarction	44,435 (44.0)	36,220 (44.0)	30,080 (43.3)	24,403 (41.7)	24,559 (39.8)
	Stroke	37,149 (57.3)	36,222 (56.1)	32,628 (55.7)	28,155 (54.1)	26,931 (53.0)
**Past medical history**						
Ischemic heart disease (%)	Myocardial infarction	10,069 (10.0)	10,887 (13.2)	10,381 (15.0)	9,713 (16.6)	9,425 (15.3)
	Stroke	6,462 (10.0)	7,274 (11.3)	6,780 (11.6)	6,341 (12.2)	5,961 (11.7)
Cerebrovascular disease (%)	Myocardial infarction	4,934 (4.9)	4,663 (5.7)	3,725 (5.4)	2,856 (4.9)	2,616 (4.2)
	Stroke	4,422 (6.8)	4,093 (6.3)	2,727 (4.7)	2,044 (3.9)	1,666 (3.3)
Heart failure (%)	Myocardial infarction	6,507 (6.4)	6,445 (7.8)	5,467 (7.9)	4,156 (7.1)	3,663 (5.9)
	Stroke	3,717 (5.7)	3,765 (5.8)	3,138 (5.4)	2,482 (4.8)	2,299 (4.5)
Coronary revascularisation (%)	Myocardial infarction	619 (0.6)	818 (1.0)	878 (1.3)	1408 (2.4)	1490 (2.4)
	Stroke	372 (0.6)	675 (1.0)	828 (1.4)	1154 (2.2)	1391 (2.7)
Cardiac devices, (%)	Myocardial infarction	343 (0.3)	433 (0.5)	436 (0.6)	458 (0.8)	573 (0.9)
	Stroke	282 (0.4)	423 (0.7)	418 (0.7)	469 (0.9)	547 (1.1)
**Deprivation (SIMD),** **quintile (%)**						
One (most deprived)	Myocardial infarction	27,438 (27.7)	21,903 (26.8)	17,959 (26.0)	14,069 (24.2)	15,079 (24.6)
	Stroke	17,357 (27.3)	16,833 (26.2)	14,958 (25.7)	12,649 (24.4)	11,988 (23.7)
Two	Myocardial infarction	24,060 (24.3)	19,993 (24.5)	16,645 (24.1)	13,585 (23.4)	14,122 (23.1)
	Stroke	15,115 (23.8)	15,343 (23.9)	13,738 (23.6)	11,856 (22.9)	11,370 (22.5)
Three	Myocardial infarction	19,904 (20.1)	16,455 (20.2)	13,846 (20.1)	11,883 (20.4)	12,278 (20.1)
	Stroke	12,717 (20.0)	12,957 (20.2)	11,621 (20.0)	10,514 (20.3)	10,136 (20.1)
Four	Myocardial infarction	15,546 (15.7)	12,993 (15.9)	11,307 (16.4)	10,288 (17.7)	10,841 (17.7)
	Stroke	10,298 (16.2)	10,470 (16.3)	9,799 (16.8)	9,079 (17.5)	9,099 (18.0)
Five (least deprived)	Myocardial infarction	12,053 (12.2)	10,272 (12.6)	9,233 (13.4)	8,326 (14.3)	8,880 (14.5)
	Stroke	8,139 (12.8)	8,586 (13.4)	8,121 (13.9)	7,650 (14.8)	7,890 (15.6)

### 3.1 Incidence of myocardial infarction and stroke

**Fig. 1.**
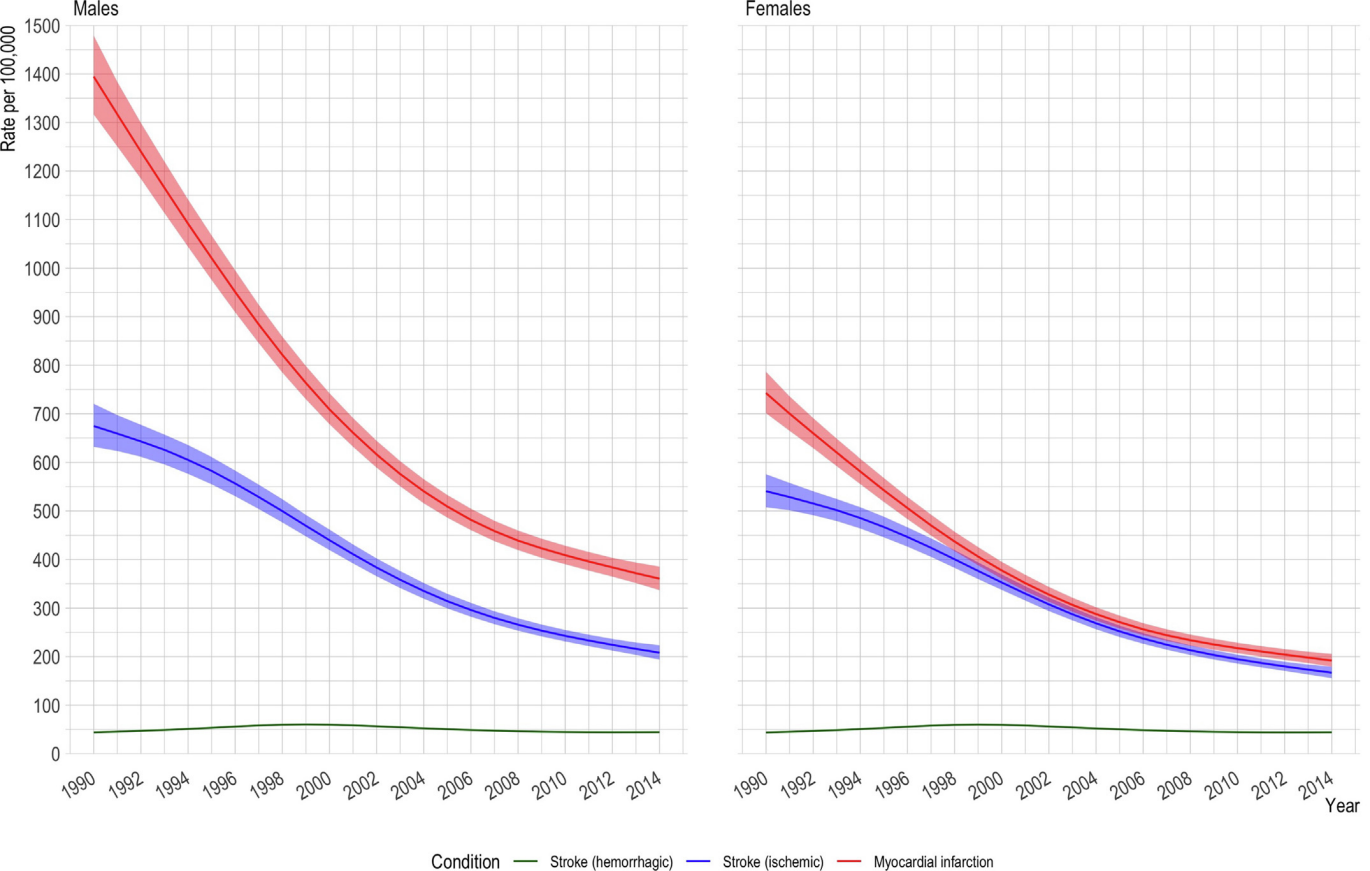
Age standardized incidence rate per 100,000 for myocardial infarction, ischemic stroke and hemorrhagic stroke.

Age-standardized incidence per 100,000 fell from 1,069 [95% confidence interval 1,024 to 1,116] to 276 [263 to 290] for myocardial infarction, and from 608 [581 to 636] to 188 [178 to 197] for ischemic stroke but remained unchanged (44 [43 to 45] to 44[44 to 45]) for hemorrhagic stroke *(****Supplementary table 4)***. Similar trends were observed in males and females ***(***Figure 1***)*** with the highest reductions observed in older age groups ***(Supplementary figure 2)***.

### 3.2 Changes in risk factors, potential impact fraction and burden

Over the 25 years, age- and sex-standardized risk factor levels reduced for systolic blood pressure (140 [139 to 140] to 129 [129 to 130] mmHg), smoking prevalence (58 [54 to 63] to 25 [24 to 26] %) and cholesterol concentrations (6.4 [6.2 to 6.6] to 5.1 [5.0 to 5.3] mmol/L). Body mass index (27.2 [27.0 to 27.4) to 28.1 [28.0 to 28.2] kg/m^2^) and diabetes prevalence (4 [3 to 5] to 9 [8 to 9] %) increased. The trends remained consistent across the majority of age and sex strata***(Supplementary figure3)***. Changes in risk factor prevalence accounted for a 74 [57-91] % and 68 [55-83] % age- and sex-standardized reduction in myocardial infarction and ischemic stroke. This was predominantly driven by reduction in systolic blood pressure for ischemic stroke and in cholesterol and smoking rates for myocardial infarction ***(***Figure 2a***).*** In contrast, we observed a 20 [16 to 26] % and 15 [11 to 21] % increase in myocardial infarction and ischemic stroke incidence attributable to changes in body-mass index and diabetes prevalence ***(Supplementary table 5)*** Differences were observed in the potential impact fractions according to age and sex. Reductions in smoking rates and increase in body-mass index had the largest impact on younger people whilst increases in diabetes prevalence predominantly impacted the elderly ***(Supplementary figure 4and table 6)***.

**Fig. 2a.**
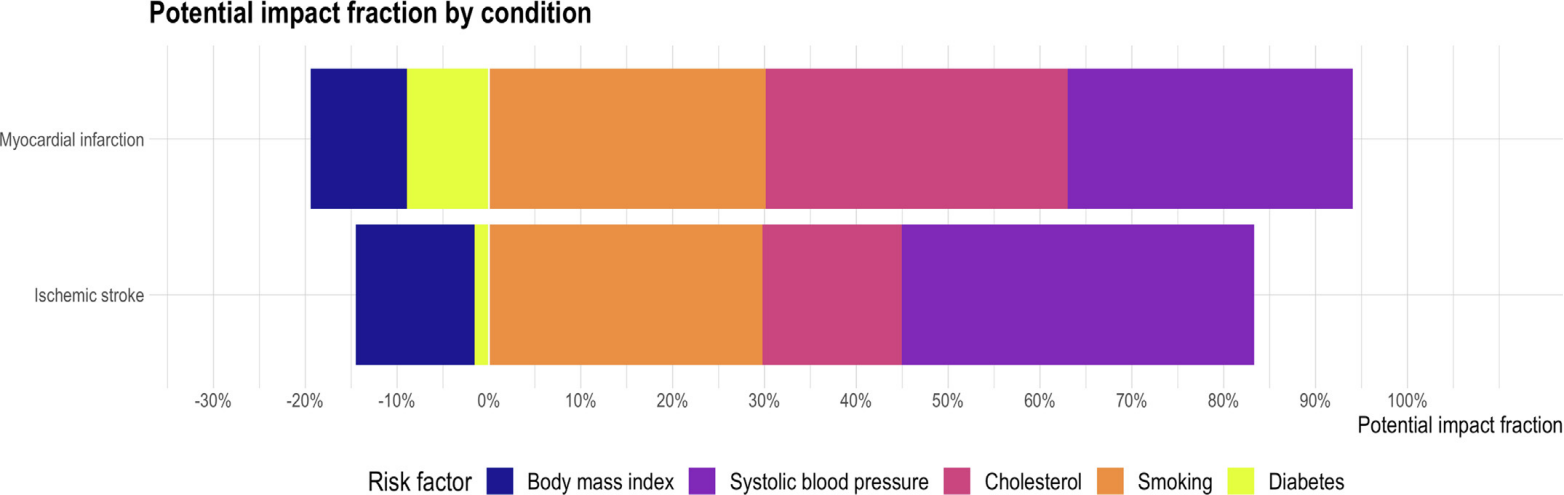
Stack barplot showing potential impact fraction for myocardial infarction and ischemic stroke by risk factor change from 1990 to 2014.

Changes in risk factors over 25 years had a larger impact on reducing burden for incident myocardial infarction (net reduction in events 34,762 [23,412 to 43,727]) than for ischemic stroke (13,247 [9,390 to 16,938])***(***Figure 2b***, Supplementary table 7).*** For myocardial infarction, a reduction in smoking rates prevented 9,362 [7,811 to 11,046] events, but the increase in diabetes prevalence contributed to 7,544 [4,769 to 10,377] additional events.

**Fig. 2b.**
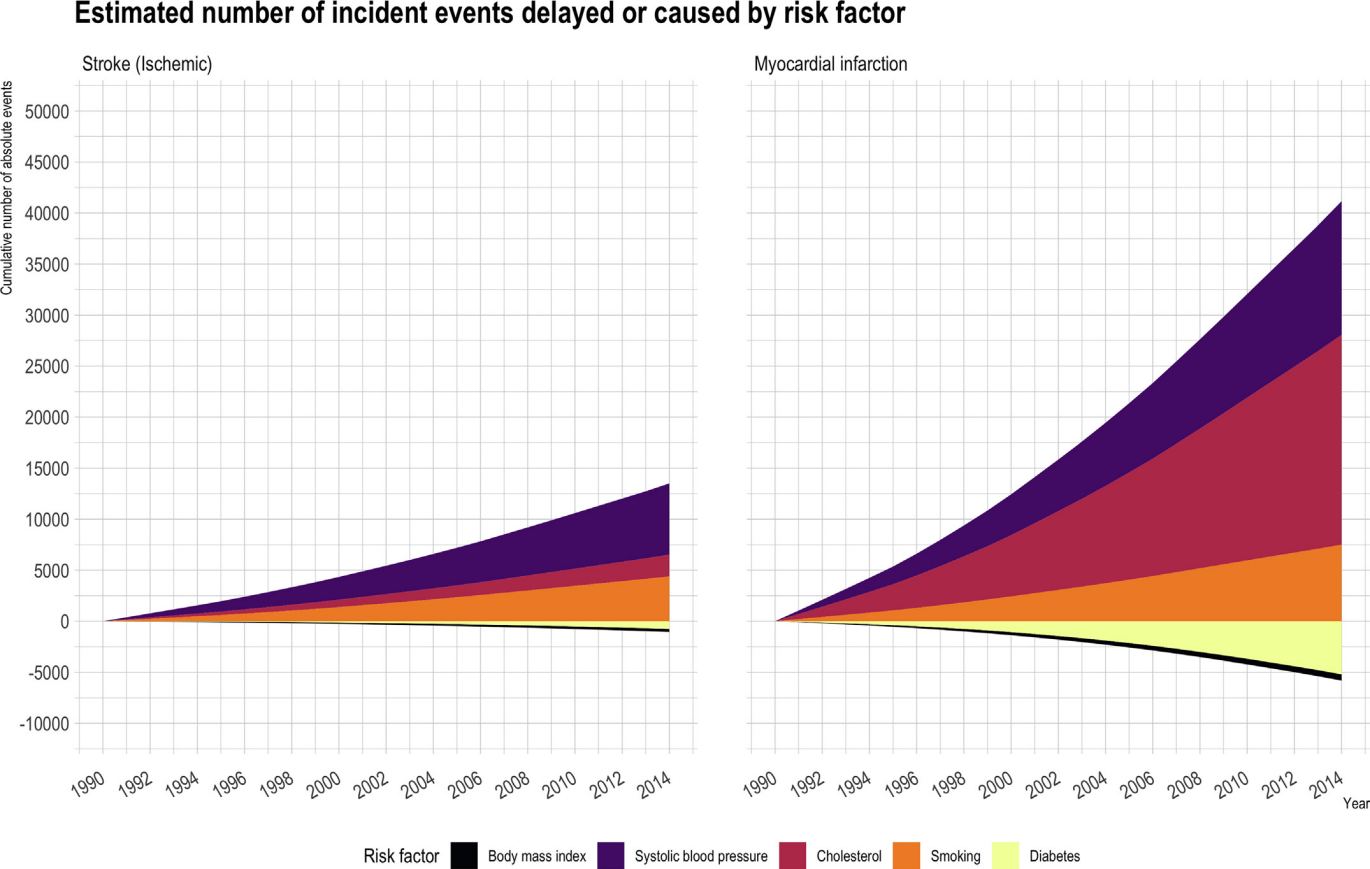
Stack plot showing absolute number of index myocardial infarction and ischemic stroke events delayed / prevented or caused by change in risk factor level.

### 3.3 Outcomes following incident cases

Crude 30-day and 3-year case-fatality rates, including index deaths, fell for myocardial infarction and ischemic or hemorrhagic stroke***(***Table 2***)*** with similar trends observed on restricting analyses to index non-fatal cases***(Supplementary table8)***.

**Table 2.** Fatal and non-fatal outcomes in patients with fatal and non-fatal index myocardial infarction and stroke stratified by 5- calendar year groups.

Outcome type	Index Condition	Outcome time	1990 to 1994	1995 to 1999	2000 to 2004	2005 to 2009	2010 to 2014
Myocardialinfarction	Myocardial infarction	30 days	1,333 (1.5)	1,486 (2.1)	1,560 (2.7)	1,501 (3.1)	2,976 (5.5)
		One year	4,581 (5.1)	3,892 (5.5)	3,816 (6.6)	3,535 (7.2)	5,550 (10.2)
		Three years	7,086 (7.9)	5,453 (7.8)	5,163 (8.9)	4,860 (9.9)	7,337 (13.5)
	Stroke	30 days	201 (0.4)	236 (0.4)	188 (0.3)	191 (0.4)	151 (0.3)
		One year	920 (1.7)	802 (1.3)	684 (1.2)	645 (1.3)	600 (1.2)
		Three years	1,710 (3.2)	1,513 (2.5)	1,268 (2.3)	1,190 (2.4)	1,175 (2.4)
Stroke	Myocardial infarction	30 days	76 (0.1)	234 (0.3)	206 (0.4)	199 (0.4)	209 (0.4)
		One year	295 (0.3)	791 (1.1)	741 (1.3)	698 (1.4)	780 (1.4)
		Three years	699 (0.8)	1,515 (2.2)	1,320 (2.3)	1,254 (2.6)	1,399 (2.6)
	Stroke	30 days	928 (1.7)	1,152 (1.9)	1,005 (1.8)	1,037 (2.1)	1,264 (2.6)
		One year	5,249 (9.7)	5,214 (8.8)	4,232 (7.7)	3,774 (7.7)	3,453 (7.1)
		Three years	7,831 (14.4)	7,618 (12.8)	6,159 (11.2)	5,405 (11.0)	5,048 (10.4)
Heart failure	Myocardial infarction	30 days	1,223 (1.4)	1,326 (1.9)	1,301 (2.2)	1,284 (2.6)	1,353 (2.5)
		One year	4,895 (5.5)	4,671 (6.7)	4,274 (7.4)	3,479 (7.1)	3,665 (6.8)
		Three years	7,267 (8.1)	6,694 (9.5)	5,837 (10.1)	4,847 (9.9)	5,105 (9.4)
	Stroke	30 days	200 (0.4)	196 (0.3)	165 (0.3)	160 (0.3)	133 (0.3)
		One year	1,206 (2.2)	1,225 (2.1)	978 (1.8)	852 (1.7)	836 (1.7)
		Three years	2,337 (4.3)	2,384 (4.0)	1,899 (3.5)	1,659 (3.4)	1,693 (3.5)
Bleeding	Myocardial infarction	30 days	142 (0.2)	192 (0.3)	297 (0.5)	443 (0.9)	538 (1.0)
		One year	854 (1.0)	897 (1.3)	1,222 (2.1)	1,457 (3.0)	1,804 (3.3)
		Three years	1,877 (2.1)	1,916 (2.7)	2,226 (3.8)	2,489 (5.1)	2,997 (5.5)
	Stroke	30 days	529 (1.0)	591 (1.0)	585 (1.1)	495 (1.0)	479 (1.0)
		One year	2,020 (3.7)	1,775 (3.0)	1,720 (3.1)	1,546 (3.1)	1,466 (3.0)
		Three years	3,406 (6.3)	2,981 (5.0)	2,939 (5.4)	2,699 (5.5)	2,620 (5.4)
Death	Myocardial infarction	30 days	39,557 (44.1)	27,735 (39.5)	19,711 (34.0)	13,752 (28.0)	10,044 (18.5)
		One year	45,899 (51.2)	32,732 (46.6)	24,544 (42.4)	18,204 (37.1)	14,528 (26.8)
		Three years	52,106 (58.1)	37,567 (53.5)	28,959 (50.0)	22,229 (45.3)	19,427 (35.9)
	Stroke	30 days	15,162 (28.0)	17,961 (30.2)	14,734 (26.9)	11,370 (23.2)	9,341 (19.3)
		One year	24,665 (45.5)	26,722 (45.0)	22,784 (41.5)	18,223 (37.1)	15,639 (32.3)
		Three years	32,049 (59.1)	33,675 (56.7)	28,986 (52.9)	23,708 (48.3)	21,205 (43.9)

**Fig. 3.**
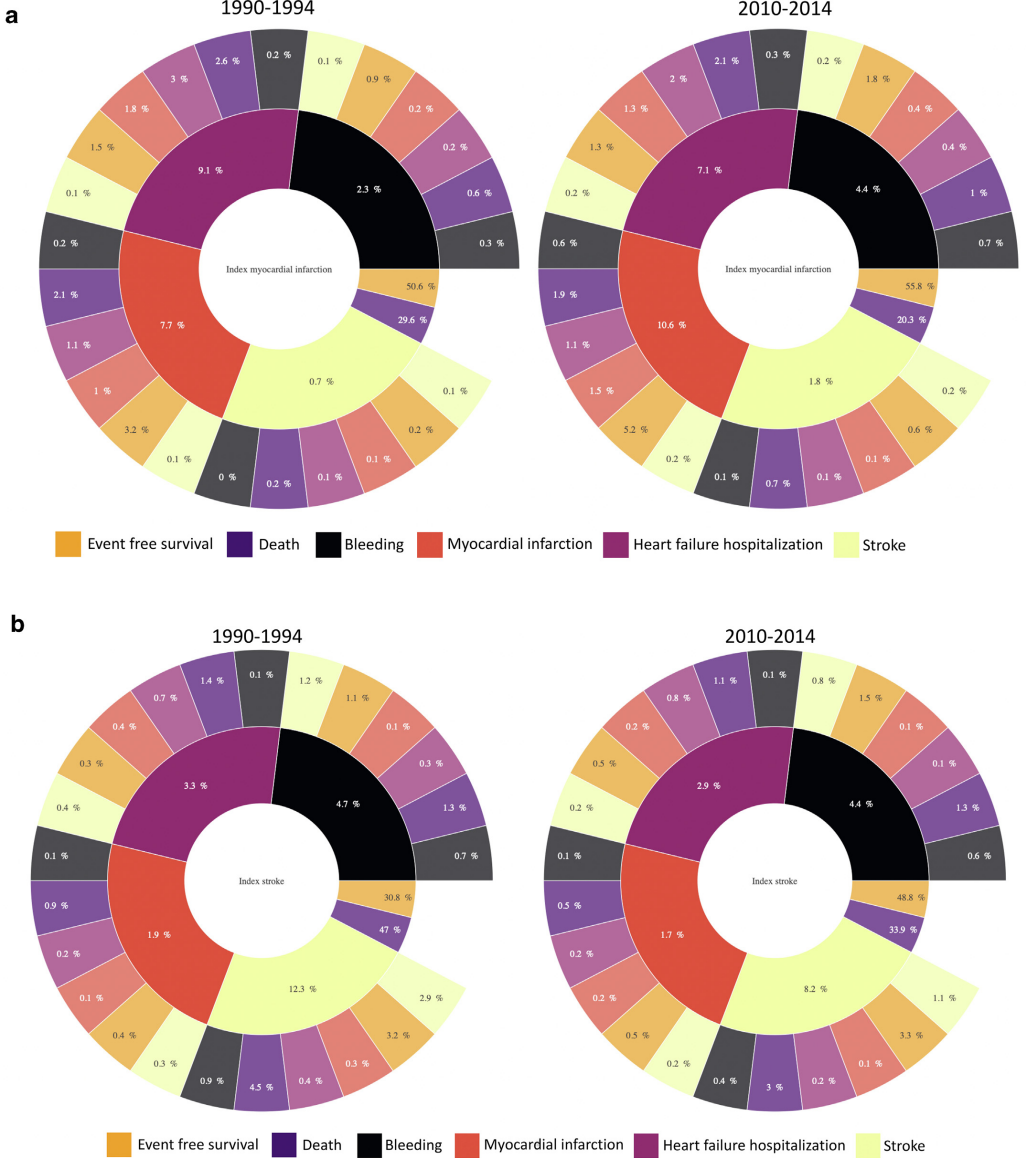
Population risks from multistate models – unscaled sunburst plots showing stratum standardized risks for the population for fatal and non-fatal sequalae following index non-fatal myocardial infarction (A) and stroke (B). *Note: A web application has been created (*https://ihwph-hehta.shinyapps.io/614967/*) to illustrate the predicted risks for up-to three levels for fatal or non-fatal subsequent events and four levels for subsequent fatal events. The web application also provides conditional probabilities of risks stratified by age, sex, deprivation, presence or absence of comorbidity and cohort period. An adjunct explanatory document has also been submitted to summarize how to use the web-application. On publication, the web application will be updated with the explanation.*

Following myocardial infarction and stroke, the risk of death with no interceding event decreased from 30% to 20% and from 47% to 34% respectively. Risk of any subsequent non-fatal event increased for myocardial infarction (from 20% to 24%) but decreased for stroke (from 22% to 17%). Following myocardial infarction, there was an increase in recurrent myocardial infarction (from 7.7% to 10.6%) and bleeding (from 2.3% to 4.4%) although heart failure hospitalisations fell (from 9.1% to 7.1%). Following stroke, the risk of recurrent stroke was reduced (from 12.3% to 8.2%) and bleeding remained unchanged (from 4.7% to 4.4%) ***(***Figure 3***)***. Risks following the index event differed when evaluated by age, sex, deprivation and comorbidity. An online interactive web application reports the standardized risk of fatal and non-fatal eventsat 3-years for index myocardial infarction and stroke by period and stratified by age, sex, deprivation, and comorbidity (https://ihwph-hehta.shinyapps.io/614967/).

## 4 Discussion

There have been substantial changes in the epidemiology of myocardial infarction and stroke over the last 25 years. The incidence of myocardial infarction and ischemic stroke has reduced 3- to 4-fold with differences in the age-standardized incidence narrowing for both men and women. We estimate that over two-thirds of these reductions can be attributed to major changes in population risk factors with substantial falls in systolic blood pressure, cholesterol concentrations and smoking rates albeit attenuated by increases in body-mass index and diabetes prevalence. Outcomes following myocardial infarction and stroke have also changed substantially. Death following the index event has decreased for both myocardial infarction and stroke, but absolute rates remain high particularly for stroke. Finally, the risk of subsequent non-fatal clinical events is diverging with rising recurrent events following myocardial infarction and falling following stroke.

The trends in myocardial infarction and stroke incidence reported here are consistent with many previous studies across industrialized countries over the last three decades. Whilst these prior studies have predominantly focused on trends in the incidence of coronary deaths[4], [5], [6], [7], [8], [9] or non-fatal stroke,[20]we observe consistent reductions in the incidence of both fatal and non-fatal myocardial infarction and stroke. Ford *et al* observed a 50% reduction in coronary deaths from 1980 to 2000 in the United States.[8] Across a similar time period, the Oxford Vascular study, with robust case ascertainment, reported a 40% reduction in non-fatal stroke.[37] In our study, the contemporaneous reductions in fatal and non-fatal myocardial infarction and ischemic stroke were even greater. Importantly, we now show that the incidence of both conditions has continued to decline in the decade that followed and the differences in the incidence narrowing.

Our analysis also showed a trend towards a reduction in social inequality. The proportion of patients with incident myocardial infarction and stroke has steadily decreased (from 28% to 25% and 27% to 24% respectively) in the most deprived group and increased (from 12% to 15% and 13% to 16%) in the least deprived group. Across the United Kingdom, McCartney et al showed that the most deprived group experienced the highest absolute reduction in incident coronary mortality but on the relative scale the rate of reductions are higher for the least deprived groups.[38]

In our study, over two-thirds of the reduction in the incidence of myocardial infarction and stroke can be attributed to changes in risk factor prevalence over 25 years. We demonstrated considerable reductions in systolic blood pressure, cholesterol and smoking, similar to changes observed in other industrialized countries.[39,40] For reductions in incident myocardial infarction, changes in systolic blood pressure, cholesterol and smoking rates contributed equally, whereas change in systolic blood pressure was the major determinant of the reduction in incident stroke. Increases in the screening and treatment of hypertension and dyslipidaemia, together with the use of more potent pharmacotherapies, alongside transformative public health efforts, such as smoking bans in public places, are likely to account for a large part of the reductions observed in our study.[39,[41], [42], [43], [44]] Whilst smoking rates fell across age groups, the greatest reductions were observed in younger persons, particularly women. Smoking increases the risk of myocardial infarction 6-fold in women, compared to 3-fold in men.[14] Stronger associations coupled with the greatest temporal reduction, made smoking the single most important factor associated with the reduction in myocardial infarctionand stroke in young women.

Whilst the majority of risk factor changes have been favorable, two exceptions are noteworthy and concerning. Increases in body-mass index and a doubling in the prevalence of diabetes has contributed considerably to incident myocardial infarction and stroke, attenuating the gains observed from better cholesterol and blood pressure control and reduced smoking rates. In our analysis, body-mass index increased in younger persons, but was unchanged in the elderly, and this contributed to an estimated increase in incident stroke and myocardial infarction by a third and quarter respectively. Whilst the prevalence of diabetes doubled, increases were more marked in the elderly, and given the stronger association between diabetes and coronary heart disease, this contributed to a 20% increase in incident myocardial infarction, with little impact on incident stroke. Overall, we estimate that across all age groups in the population, the increasing prevalence of diabetes contributed to as many incident myocardial infarctions as were prevented by the reduction in smoking. Public health efforts now need to urgently focus on managing obesity and diabetes to further curb cardiovascular disease.[45,46]

In parallel to these major changes in cardiovascular risk factors and the incidence of myocardial infarction and stroke, our analysis also demonstrate important changes in morbidity and mortality in those who survive the index event. Across the 25-year period,the case-fatality rate declined from approximately 1 in 3 to 1 in 5 following myocardial infarction, and from 1 in 2 to 1 in 3 following stroke. Improving survival for both these conditions likely reflects improvements in secondary prevention[47] and antithrombotic[48]pharmacotherapy, introduction of acute stroke units,[49] and coronary revascularisation strategies.[50]However, as illustrated in our online interactive web application, the absolute risks of death across the population and across age, sex and deprivation strata remain substantially higher for stroke compared to myocardial infarction. In addition to the higher case-fatality rate following stroke, there has been a substantial narrowing in the differences between the incidence of myocardial infarction and stroke since 1990. Indeed, the incidence rates of myocardial infarction and ischemic stroke are now identical in women. As a consequence, stroke now contributes to an increasingly greater burden of acute cardiovascular events and healthcare provision needs to recognize this.

In contrast to the consistent reductions in case fatality for both myocardial infarction and stroke, the risk of non-fatal sequelae is diverging with an increase in recurrent events following myocardial infarction and a decrease following stroke. Following myocardial infarction, the risk of recurrent myocardial infarction increased from 8% to 11% likely reflecting changes in the clinical definition,the introduction of more sensitive troponin assays[51,52] and improving survival from the index event. The observation that subsequent bleeding rates doubled is likely to be a consequence of the increasing use of more potent anti-platelet and anti-thrombotic therapies.[53] The risk of recurrent stroke was reduced without change in bleeding rates, perhaps suggesting the management of other risk factors is responsible for improved outcomes.[15]

There are several potential strengths to our study. First, our approach ensured complete follow-up in those patients who remained resident in Scotland during the study period. Similar approaches have been used to deliver randomized clinical trials[54,55] and cohort studies.[32,56] Second, our population consists of consecutive patients with myocardial infarction or stroke, avoiding selection bias and improving generalizability. Third, national administrative datasets in Scotland have been operational for decades, with mature, high quality and consistent data.[57] Diagnostic coding for both stroke and ischemic heart disease have been validated against case note reviews and audit data showing good accuracy.[58], [59], [60]

Several limitations need to be highlighted. First, when estimating the potential impact fraction, we assume that each risk factor is mutually exclusive.[61] In reality, both myocardial infarction and stroke occur as a consequence of numerous causal risk factors, rather than a single cause.[61] As such the estimates in the reduction in incidence for each individual risk factor is likely to be an over-estimate. Second, we did not consider the effect of lag times between changes in risk factor levels and incident disease in our analysis. The impact of smoking cessation is likely to be immediate, whereas the effects of weight gain and diabetes may not be observed for many years. Third, the population of Scotland is predominantly white, with small numbers of minority ethnic groups thereby precluding stratified analyses by ethnicity. Fourth, our study predominantly focuses on biological and behavioural risk factors to the exclusion of social ones. Finally, other outcomes following myocardial infarction and stroke are important, such as requirements for social and personal care, and these are not captured by our administrative data. Further, research is now needed to evaluate the role social risk factors and its influence on changes in risk factor levels and subsequent incidence of fatal and non-fatal cardiovascular disease.

Our findings have implications for public health policy and healthcare provision. Substantial reductions in the incidence of both myocardial infarction and ischemic stroke can be achieved by addressing biological and behavioural risk factors. These observations have major implications for countries in low- and middle-income settings where rates of hypertension[39] and dyslipidaemia[62] are rising as well as the residual risk from persistent uncontrolled risk factors in high-income countries. Despite the overall gains, we also highlight the increasing influence of obesity and diabetes on acute cardiovascular events, which have implications for all countries given that both risk factors are risingworldwide.[62,63] Finally, differences in the burden of myocardial infarction and stroke are narrowing, particularly in women, and monitoring these trends is essential for the planning of healthcare provision in the decades that follow.

In conclusion, our analysis highlights a considerable decline in incident myocardial infarction and stroke with over two-thirds of the reduction attributable to favorable changes in risk factor profiles, but increasing obesity and diabetes continue to contribute to the clinical burden.Important changes in the risk of subsequent events following index stroke and myocardial infarction have occurred. Whilst initial case-fatality following stroke has reduced considerably, absolute rates remain high.

**Contributors:** ASVS conceived the study. ASVS and DM designed the study. ASVS, DM, DC, JP, FA and KKL were involved in the analysis of the study. ASVS drafted the first version of the manuscript. All authors provided critical input into the paper.

**Declaration of interest:** JL has is on the Advisory Board and has received Research Consultancy fees from Novo Nordisk**.** This study was funded by the British Heart Foundation through an Intermediate Clinical Research Fellowship (FS/19/17/34172) and a Clinical Lecturer Starter Grant from the Academy of Medical Sciences. DAM is supported by a Wellcome Trust Intermediate Clinical Fellowship (201492/Z/16/Z). SVK is funded by a NRS Senior Clinical Fellowship (SCAF/15/02), the Medical Research Council (MC_UU_00022/2 and the Scottish Government Chief Scientist Office (SPHSU17). DC is funded by an unrestricted gift from Baillie Gifford. DEN is supported by the British Heart Foundation (CH/09/002, RG/16/10/32375, RE/18/5/34216) and Wellcome Trust (WT103782AIA). All other authors have no conflicts to declare.

**Data sharing:** We are unable to share individual patient level data including covariate data but this is available on application to the Information Services Division at Public Health Scotland and subject to approval from the NHS Scotland Public Benefit and Privacy Panel for Health and Social Care. Individual participant level on risk factors across the population surveys are available from the UK data service and accessible via https://www.gov.scot/publications/scottish-health-survey-dataset-information/.

## Appendix Supplementary materials

Image, application 1

Image, application 2
