# Supplementary material for: Clinical burden, risk factor impact and outcomes following myocardial infarction and stroke: A 25-year individual patient level linkage study
Source: Lancet Reg Health Eur. 2021 Jun 16;7:100141. doi: 10.1016/j.lanepe.2021.100141 (PMC8351196; doi:10.1016/j.lanepe.2021.100141)
Supplement: Supplementary file 1 [file mmc1.docx]

**Research in Context**

Evidence before this study: We searched Ovid Medline (from 01/01/1995 to 01/08/2020) for search terms ‘cardiovascular risk factors’, ‘incidence’, ‘myocardial infarction’, ‘stroke’ and ‘outcomes’. We several studies including systematic reviews evaluating trends in incident myocardial infarction or stroke. Several studies evaluated impact of changes in risk factors on coronary death. We found no large contemporary nationwide studies comparing the clinical burden of stroke and myocardial infarction, evaluating the impact of population changes in risk factors on fatal and non-fatal disease and the short- and longer-term fatal and non-fatal clinical sequalae following index event.

Added value of this study: In a large contemporary nationwide patient level linkage study we show that substantial reductions in myocardial infarction and ischemic stroke incidence are associated to reductions in systolic blood pressure, smoking and cholesterol but attenuated by increases in body mass index and diabetes prevalence. Importantly, we now show that the that in addition to incidence of both myocardial infarction and stroke declining, the difference in the incidence has also narrowed. Deaths following the index event decreased for both myocardial infarction and stroke, but rates remained substantially higher for stroke. The clinical burden of stroke, both in terms of incidence and case-fatality, now contributes to a greater burden of acute cardiovascular disease.

Implications of all the available evidence: Substantial reductions in the incidence of both myocardial infarction and ischemic stroke can be achieved by addressing biological and behavioural risk factors. These observations have major implications for countries in low- and middle-income settings where rates of hypertension and dyslipidaemia are rising as well as the residual risk from persistent uncontrolled risk factors in high-income countries. We also highlight the increasing influence of obesity and diabetes on acute cardiovascular events, which have implications for all countries given that both risk factors are rising worldwide. Finally, differences in the burden of myocardial infarction and stroke are narrowing, particularly in women, and monitoring these trends is essential for the planning of healthcare provision in the decades that follow.
