# Supplementary material for: Clinical burden, risk factor impact and outcomes following myocardial infarction and stroke: A 25-year individual patient level linkage study
Source: Lancet Reg Health Eur. 2021 Jun 16;7:100141. doi: 10.1016/j.lanepe.2021.100141 (PMC8351196; doi:10.1016/j.lanepe.2021.100141)
Supplement: Supplementary file 2 [file mmc2.docx]

**Reviewer #1: This paper is the analysis of temporal trends in myocardial infarction (AMI) and stroke incidence in Scotland and the associated changes in risk factor levels. The observation that obesity and diabetes are eroding the gains attributable to improvements in other risk factor levels is important, not just for Scotland. A word appears to be missing on page 8, "Risks following the index event...", and page 20, "...we show substantial that..." Reference 53 needs to be corrected.**

*We thank the reviewer for the comments provided. We have now revised the sentences and corrected reference 53.* **Reviewer #2: This study describes trends in the incidence and outcomes of myocardial infarction (MI) and stroke, and examines how trends were influenced by changes in cardiovascular risk factors. It included all patients in Scotland who were 30 years of age or older with MI or stroke over a 25 year period beginning in 1990. The study drew from a variety of individual and population level data sources. Index MI or stroke events were measured at the patient level along with the patient's sex, comorbidity and area-based measure of deprivation. In addition, the study measured hospital episodes prior to and after the index event as well as deaths with or without hospitalizations. The main outcomes after the index event were morality and non-fatal events consisting of heart failure, stroke, myocardial infarction and bleeding. Risk factors were measured at the population level from periodic national health surveys. Risk factors included systolic blood pressure, total cholesterol, body mass index and the prevalence of smoking and diabetes mellitus. The study found a substantial decline in the number and standardized incidence of MI and stroke over the 25-year period. Concurrently, there was a substantial reduction in systolic blood pressure, smoking and cholesterol. According to estimates from study, the decline in these population risk factors accounted for a 74% reduction in MI and 68% reduction in ischemic stroke. However, gains from these risk factor reductions were offset by increases in other risk factors of body-mass index and diabetes prevalence. Outcomes after the index events also showed some improvements. Following MI, the risk of death decreased 30% to 20%, but non-fatal events increased from 20% to 24%. Following stroke, the risk of both death (47% to 34%) and non-fatal events (22% to 17%) decreased.

The study addressed an important gap in the literature on longitudinal trends in MI and stroke and associated changes in risk factors in a large population-based study over an extended period. The study leveraged a creative synthesis of data from administrative records, surveys, and published sources. The findings are compelling and they have important implications for public health. The manuscript is well written and the tables and figures are informative.**

*We thank the reviewer for comments on our analyses.*
 **Despite these considerable strengths, I have several concerns.

1. Several limitations are noted by the authors: the method for estimating the potential impact fraction likely over-estimated the risk reduction impact of the individual risk factors; lag times between changes in risk factor levels and incident disease were not accounted for; generalization of the findings is limited because the population of Scotland is predominantly white, with small numbers of minority ethnic groups; the study predominantly focuses on biological and behavioral risk factors to the exclusion of social ones; and important societal outcomes such as requirements for social and personal care are not captured by the administrative data.**

*We agree and as the reviewer highlights there are several limitations in our work that should be taken into consideration when interpreting our data. All these points, including those of generalizability, have been acknowledged in the manuscript. We have now added some text to highlight these issues as important topics for further research too (Page 14, Paragraph 2).*

**2. The selection of index MI and stroke events should be clarified. The study tracks index admission events and other MI or stroke admissions in each year (Supplementary Materials, Lookback Period). In calculating incidence during a year, was any admissions for MI or stroke counted, or only the index admission event? If only the index event, then explain why other admissions for MI or stroke were not considered. Wouldn't singling out index admissions lead to an underestimate the annual incidence rate?**

*We apologise if this was not clear in our manuscript. We confirm that index myocardial infarction and stroke were treated separately. As such if a patient was to have an index myocardial infarction (using a 5 year look back period) and was to subsequently suffer an index stroke – that individual would be counted in the numerator for both calculating incident myocardial infarction and stroke.*

*If a participant was however to suffer an index myocardial infarction and subsequently suffer a second myocardial infarction, then only the first event will be counted as the index event (except in the scenario that the second myocardial infarct occurred more that 5 years after the index infarct - the rationale for the 5-year look back).*

*We have clarified this point in the revised manuscript (page 5, paragraph 1 and page 7, paragraph 1).*

**3. The absence of social risk factors is mentioned as a limitation. Could the Scottish Index of Multiple Deprivation (SIMD) be considered a social risk factor? This measure does not figure as prominently in the findings as age and sex stratification. Could changes in the social deprivation measure be incorporated into the risk factor analysis, or be given greater attention as a moderator of MI and stroke incidence rates? In Table 1, the percentage distribution of MI and stroke appears to be decreasing among the most deprived and increasing among the least deprived. What can be made of this finding?**

*This is an important point. Specific social risk factors at an individual level (social support, access to care etc) are not captured by the administrative datasets. SIMD is based on 7 postcode-based domains including income, employment, health, education, skills and training, geographic access to services, crime and housing. As such we agree with the reviewer that SIMD could be considered as a surrogate for social risk factors.*

*Our current approvals for access to individual patient level data have now been terminated and additional approvals have now been sought to a) extend the cohort period for incident myocardial infarction and stroke to 2021 and b) evaluate trends in treatment and management differences by SIMD. These analyses are planned as a separate manuscript to specifically concentrate on SIMD but data access, cleaning and analysis will not be possible for some time – at least in part since the safe haven is prioritising urgent COVID-19 projects. As such we are unable to provide further stratified analyses by SIMD in our current manuscript.*

*Specific to the reviewers comments please see our responses below:*

*Outcomes: We have already incorporated the SIMD as a covariate in evaluating outcomes following myocardial infarction or stroke. SIMD was used as a covariate in the multi-state models constructed to evaluate outcomes following index myocardial infarction or stroke.*

*Population attributable fraction: Whilst our population risk factor level data, from the Scottish health surveys, were estimated stratified by calendar time, age and sex, we did not harmonise SIMD data. We were therefore unable to estimate prevalence rates of risk factor by SIMD in addition to age, sex and calendar time.*

*Incidence: The main scope of our current analysis was to evaluate the* *overall trends in the incidence, the population attributable fraction and changes in burden and outcomes following index event. Given the already large amount of data presented, we felt that it would be difficult to integrate a fourth dimension for social inequality and therefore did not analyse the data by SIMD strata (see point above).*

*Decrease in social inequality: The reviewer is correct and health inequalities seem to have decreased over the last 25 years. This can be seen from Table 1. We have now discussed this point in more detail in the revised manuscript (page 10, paragraph 2).*

**4. In estimating outcomes after the index event, how were competing or semi-competing risks addressed? For example, mortality is a competing risk for subsequent non-fatal events. Longer survival could contribute to increased risk of non-fatal events, perhaps accounting for the increase in non-fatal events after the index MI.**

*To specifically tackle competing events, we constructed multi-state models which allow for several possible events for a single individual. We agree with the reviewer that longer survival following index event over time is likely to be one of the reasons why rates of non-fatal events have increased or remained unchanged instead of decreasing.*

*We have illustrated these risks in an online application (available* [*here*](https://ihwph-hehta.shinyapps.io/614967/)*). As an example, the risk of subsequent death or non-fatal myocardial infarction in a 65-year-old male (no comorbidities and deprivation score 1) was 24% and 8% respectively following a non-fatal index myocardial infarction in the 1990-1994 period. In the 2010-2014 period the risk of subsequent death nearly halved (14%) whilst risk of subsequent non-fatal myocardial infarction increased to 13%. We have discussed the observations seen in the manuscript (page 12, paragraph 3).* **Reviewer #3: The authors attempted to investigate the impact of population changes in risk factors over 25 years on incident myocardial infarction and stroke so they report on changes in risk of subsequent fatal events, and single and multiple non-fatal events following index non-fatal myocardial infarction or stroke. I believe that the authors have been successful in achieving their objectives.
It is a very good article, very well organized. It has a very appropriate design and they use statistical methods that allow them to achieve their objectives. The discussion is very well done and the limitations very well discussed.**

*We thank the reviewer for the comments on our manuscript.*
